# Unusually High Risks of COVID-19 Mortality with Age-Related Comorbidities: An Adjusted Meta-Analysis Method to Improve the Risk Assessment of Mortality Using the Comorbid Mortality Data

**DOI:** 10.3390/idr13030065

**Published:** 2021-08-08

**Authors:** Andrew Antos, Ming Lai Kwong, Timothy Balmorez, Alyssa Villanueva, Shin Murakami

**Affiliations:** Department of Basic Sciences, College of Osteopathic Medicine, Touro University California, 1310 Club Drive, Mare Island, Vallejo, CA 94592, USA; aantos@student.touro.edu (A.A.); mkwong4@student.touro.edu (M.L.K.); tbalmore@student.touro.edu (T.B.); alyssa.villanueva2@tu.edu (A.V.)

**Keywords:** age-related comorbidity, COVID-19, mortality, risk assessment

## Abstract

Background: The pandemic of Coronavirus Disease 2019 (COVID-19) has been a threat to global health. In the US, the Centers for Disease Control and Prevention (CDC) has listed 12 comorbidities within the first tier that increase with the risk of severe illness from COVID-19, including the comorbidities that are common with increasing age (referred to as age-related comorbidities) and other comorbidities. However, the current method compares a population with and without a particular disease (or disorder), which may result in a bias in the results. Thus, comorbidity risks of COVID-19 mortality may be underestimated. Objective: To re-evaluate the mortality data from the US and estimate the odds ratios of death by major comorbidities with COVID-19, we incorporated the control population with no comorbidity reported and assessed the risk of COVID-19 mortality with a comorbidity. Methods: We collected all the comorbidity data from the public health websites of fifty US States and Washington DC (originally accessed on December 2020). The timing of the data collection should minimize bias from the COVID-19 vaccines and new COVID-19 variants. The comorbidity demographic data were extracted from the state public health data made available online. Using the inverse variance random-effects model, we performed a comparative analysis and estimated the odds ratio of deaths by COVID-19 with pre-existing comorbidities. Results: A total of 39,451 COVID-19 deaths were identified from four States that had comorbidity data, including Alabama, Louisiana, Mississippi, and New York. 92.8% of the COVID-19 deaths were associated with a pre-existing comorbidity. The risk of mortality associated with at least one comorbidity combined was 1113 times higher than that with no comorbidity. The comparative analysis identified nine comorbidities with odds ratios of up to 35 times higher than no comorbidities. Of them, the top four comorbidities were: hypertension (odds ratio 34.73; 95% CI 3.63–331.91; *p* = 0.002), diabetes (odds ratio 20.16; 95% CI 5.55–73.18; *p* < 0.00001), cardiovascular disease (odds ratio 18.91; 95% CI 2.88–124.38; *p* = 0.002), and chronic kidney disease (odds ratio 12.34; 95% CI 9.90–15.39; *p* < 0.00001). Interestingly, lung disease added only a modest increase in risk (odds ratio 6.69; 95% CI 1.06–42.26; *p* < 0.00001). Conclusion: The aforementioned comorbidities show surprisingly high risks of COVID-19 mortality when compared to the population with no comorbidity. Major comorbidities were enriched with pre-existing comorbidities that are common with increasing age (cardiovascular disease, diabetes, and hypertension). The COVID-19 deaths were mostly associated with at least one comorbidity, which may be a source of the bias leading to the underestimation of the mortality risks previously reported. We note that the method has limitations stemming primarily from the availability of the data. Taken together, this type of study is useful to approximate the risks, which most likely provide an updated awareness of age-related comorbidities.

## 1. Introduction

COVID-19 is an infectious disease caused by severe acute respiratory syndrome coronavirus 2 (SARS-CoV-2) [1]. It has infected over 82 million people worldwide and over 19 million people in the United States (John Hopkins University Corona Virus Online Map) [2] with cases increasing daily. As of 30 December 2020, over 1.8 million people have died from COVID-19 globally, and 341,313 deaths are currently attributed to COVID-19 in the US [2].

The Centers for Disease Control and Prevention (CDC) has published a list of medical conditions that increase the risk of severe illness from COVID-19 [3]. These comorbid diseases have two tiers; the first tier has 12 conditions that “are (an) increased risk of severe illness from the virus that causes COVID-19”; and the second tier has 11 conditions that “might be at an increased risk for severe illness from the virus that causes COVID-19” [4]. The CDC listed 125 publications for the evidence [4] (updated 30 November 2020; Accessed 28 March 2021). Our rationales are the following. Firstly, the vast majority of them use data from other countries or mixtures of different countries. The US has a diverse population, which COVID-19 has impacted disproportionately compared to the rest of the world; therefore, the risk of COVID-19 should be adjusted based on the US population. Secondly, the current risk assessment of COVID-19 mortality compares a population with and without a particular disease (or disorder). If the vast majority of the COVID-19 deaths are expected to be with comorbidities, the control population without a particular disease would include the other comorbidities, resulting in a possibility of underestimation of the mortality risks. We reasoned that the control without pre-existing comorbidities should be appropriate as a control population for the mortality risk assessment. To reduce unexpected bias from the incomplete US-State data, we focused on the mortality data as well as incorporated the format of the systematic review. Our objective is to re-evaluate the mortality data from US States and estimate the odds ratios of death by major comorbidities with COVID-19. To do so, we incorporated the control population with no comorbidity reported and assessed the risk of COVID-19 mortality with a comorbidity. This study provides a new index that provides an overview of the COVID-19 deaths associated with comorbidity. 

## 2. Methods

### 2.1. Eligibility Criteria, Information Sources and Study Selection

We followed the preferred reporting items for systematic reviews and meta-analyses protocols (PRISMA) statement [5]. The criteria for eligibility is to have comorbidity data made available to the public. The US comorbidity data was searched on PubMed and Scopus for literature search and UpToDate for the clinical overview, using the keywords, “COVID 19 AND Comorbidity”, “COVID 19 AND Comorbidities AND United States”, “COVID 19 AND Comorbidities AND United States AND Mortality”, for example. For US states and CDC data, fifty state health department websites, the Washington DC website, and the CDC website was directly accessed; the links of a total of 52 websites searched are shown in the Supplementary Material 1. All the data were collected from the websites, links, and search functions embedded on the webpages (originally accessed on June 2020; Updated December 2020). Data extraction from reports was made directly from the webpage and duplicated. The webpage links, including the states, are listed in Supplementary Material 2. Confirmation of the data was made independently by two investigators. 

### 2.2. Data Collection and Risk of Bias Assessment

The Alabama, Louisiana, Mississippi, and New York state department websites update the death tolls for their respective states. We referenced the Alabama [6], Mississippi [7], and New York data [8], which were updated on 29 December 2020. We retrieved Louisiana’s data on 17 December 2020; the Louisiana data were dated on 31 March 2020 [9]. The data surrounding deaths due to COVID-19 in Alabama, Louisiana, Mississippi, and New York were separated and, additionally, summed for analysis. Deaths that included a comorbidity were separated by their respective state. Deaths that did not suffer from a comorbidity were also separated by state. To estimate the number of comorbidities in Louisiana, we multiplied the percentages with the total number of deaths since their data only provided a comorbidity percentage. We were unable to input the decimal values from the percentage calculations for Louisiana’s numerical fields. Thus, we rounded up if the tenths decimal place was equal to or greater than five and rounded down if the tenths decimal place was less than five. For New York, we also subtracted the total deaths with a comorbidity from the total deaths from COVID-19 to deduce how many COVID-19 deaths did not have a comorbidity. The risk of bias was independently tested by two investigators. We used the ROBINS-I tool (risk of bias in non-randomized studies of interventions) [10]. 

### 2.3. Synthesis of Results and Summary Measures

To calculate the odds ratios, we used the software RevMan5 (Cochrane reviews) [11]. The random-effects method was also used to calculate the odds ratios. The inverse variance method was used since it is a common and simple mode to conduct a meta-analysis. “The inverse variance method is so named because the weight given to each study is chosen to be the inverse of the variance of the effect estimate (i.e., one over the square of its standard error). Thus, larger studies, which have smaller standard errors, are given more weight than smaller studies, which have larger standard errors. The inverse-variance random-effects model has been commonly used in the COVID-19 meta-analysis studies, for example, [12,13]. This choice minimizes the imprecision (uncertainty of the pooled effect estimate).” Statistical heterogeneity is measured as I^2^ > 0. We modified the RevMan5 guideline [11] and used the following modified heterogeneity interpretations: 0% to 30% “might not be important”, 30% to 40% “may be no to moderate heterogeneity”, 40% to 50% “may represent moderate heterogeneity”, 50% to 60% “may be moderate to substantial heterogeneity”, 60% to 75% “may represent substantial heterogeneity”, 75% to 90% “may be substantial to considerable heterogeneity” and 90% to 100% “is considerable heterogeneity”. The “thresholds for the interpretation of I^2^ can be misleading since the importance of inconsistency depends on several factors.” These observed factors include the “magnitude and direction of effects” and “the strength of evidence for heterogeneity (e.g., *p*-value from the Chi-squared test, or a confidence interval for I^2^).” 

We organized the ten comorbidities between the states to standardize them for analysis as follows. Hypertension was documented in Mississippi and New York, which was included to estimate odds ratios of hypertension. For cardiovascular disease (CVD), New York listed detailed diseases with CVD pathologies (atrial fibrillation, coronary artery disease, and stroke comorbidity) [9] and, thus, they were included under the term CVD. Three states showed the data for immunocompromised conditions, liver disease, and neurological disease odds ratios; and two states for the chronic kidney disease and obesity odds ratio figures. For chronic kidney diseases (CKD) and renal disease (RD), CKD is a part of renal disease (RD), and thus RD data included CKD; nonetheless, we did perform the analysis of CKD. The WHO designates stroke as a neurological disorder [14] and the CDC designates it as a cardiovascular or cerebrovascular disease [3]. We followed the CDC designation and, thus, included stroke data into the cardiovascular disease odds ratios. New York had more detailed designations than other States, and we treated the New York data as follows. Dementia data were included in neurologic disease, and COPD was included in lung disease. In this study, neurological disease was called neurologic disease for the consistency of disease designations. Despite the designations by the CDC, the four states also did not distinguish the diabetes types (type 1, type 2, and other types of diabetes) and are shown as diabetes.

## 3. Results

### 3.1. Study Selection and Characteristics

**Figure 1:**
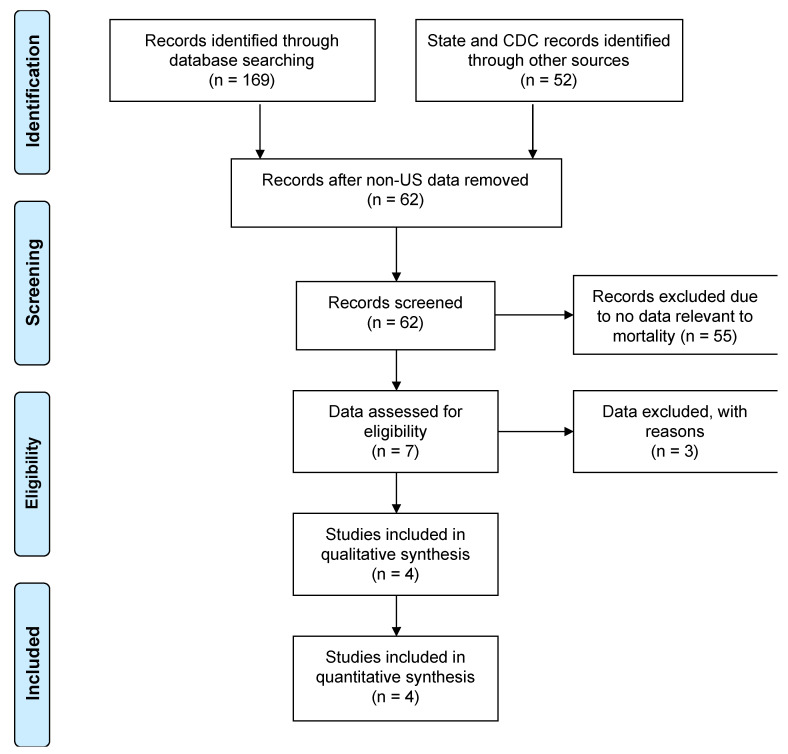
A flow diagram of this study, originally accessed on June 2020. The PRISMA template for researchers [5] was modified and used.

We modified the assessment procedure of the COVID-19 mortality data and used the control population with no comorbidity (Methods). We focused on mortality data, since the outcomes from the COVID-19 test results could be influenced by different testing systems and by how they were administered and recorded. For this reason, we excluded the COVID-19 test results. Similarly, we excluded hospitalization that may contribute to a bias of the risk assessment (Supplementary Material 3). The US comorbidity data was searched on the PubMed and Scopus websites. The results from queries resulted in a total of 1484 publications, narrowed down to 169, which included a total of 10 observational studies and case reports. The studies/reports were reviewed, and data regarding deaths from patients with and without a comorbidity in the US were not found (Accessed June 2020). Thus, the CDC and State websites were directly accessed for provisional comorbidity data; the links were listed in Supplementary Material 1. The PRISMA flow diagram in June 2020 is shown in Figure 1. The websites examined were a total of 52. The CDC had a provisional dataset of “comorbidities and other conditions”; the dataset was dominated by “pneumonia and influenza”, “respiratory failure”, and “hypertensive diseases” [15], which were mixed with COVID-19 symptoms, causes of deaths, and pre-existing conditions. Thus, we did not use the CDC dataset. 

**Figure 2:**
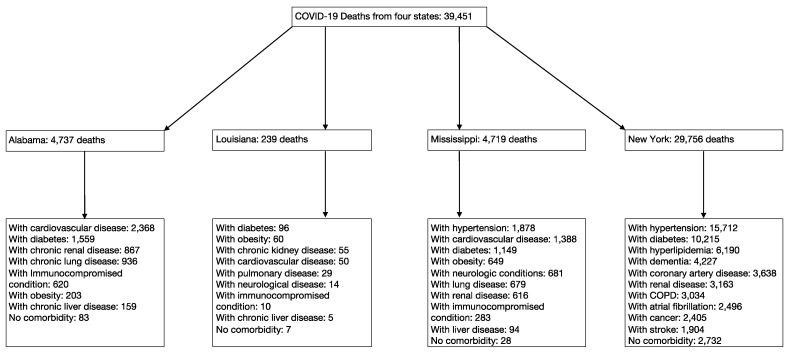
A total number of COVID-19 deaths first separated by state and then by comorbidity. A list of COVID-19 deaths was identified from a total of four US states in 2020—Alabama, Louisiana, Mississippi, and New York.

**Figure 3:**
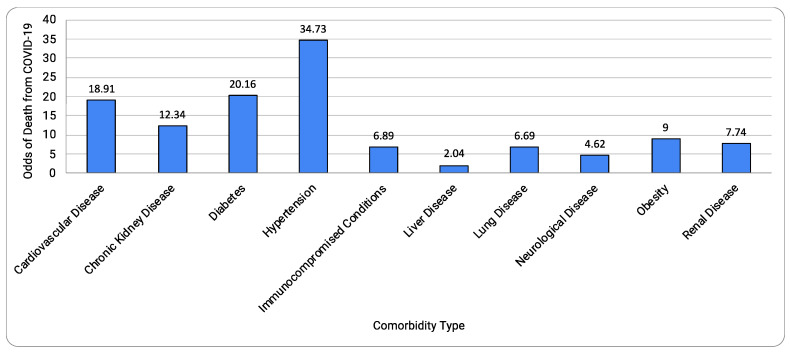
Summary of the mortality risks by COVID-19 comorbidities.

**Figure 4:**
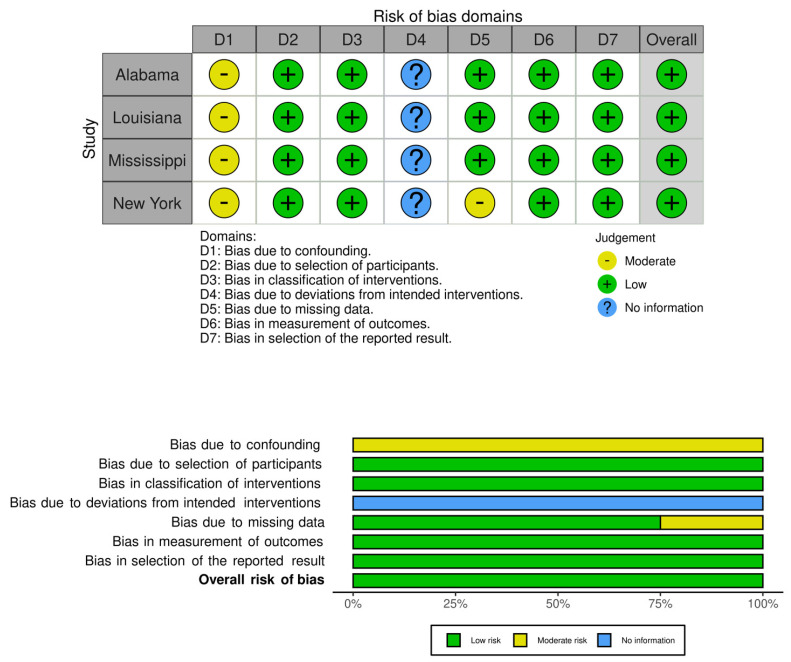
Assessment of the risk of bias using ROBINS-1. Shown are the traffic light plot (**above**) and the summary plot (**below**). We used comorbidity as an intervention and death as an outcome. No information (Blue) is not applicable to this study. We used a visualization tool as described in [17].

We collected all the data available, which was originally accessed in June 2020 and updated on December 2020 by searching the public health websites of fifty states and Washington DC in the US. Six states provided comorbidity data: Alabama, Louisiana, Mississippi, New York, Georgia, and Pennsylvania. Pennsylvania was not included in our results because the state department reported incomplete data—38% of deaths did not include comorbidity data (Pennsylvania Department of Health) [16]. We found data from a study conducted in the state of Georgia, but this study did not list the deaths by comorbidity and, thus, was not included in this study. We have compiled and organized the ten comorbidities between the states to standardize them for analysis (Table 1). The demography is summarized in Supplementary Material 2. More details are described in the Methods section. A total of 39,451 COVID-19 deaths were found in four states—Alabama, Louisiana, Mississippi, and New York. The results of the total deaths and other separations by the state are summarized in Figure 2. Each of the pre-existing comorbidities associated with COVID-19 are summarized in Figure 3. The risk of bias within studies was low to modest (Figure 4). New York accounts for most of the total COVID-19 deaths in this study, which may create a bias by dominating the result. To minimize the bias, we used the random-effects models to control the results of the odds ratio. As a comparison, New York accounted for 75.4% of the total deaths in the study, while Mississippi accounted for 11.9%. Alabama accounted for 12%, while Louisiana accounted for 0.6% of the total deaths in the study. Therefore, the random-effects model helped mitigate some of the bias from New York. 92.8% of the COVID-19 mortality was seen in patients with at least one comorbidity.

### 3.2. Synthesis of Results and Risk of Bias Assessment

Table 1 and Figure 3 summarize the results. Nine out of ten comorbidities combined to show a surprisingly high risk of death due to COVID-19, 1113 times higher (odds ratio, 1113.59; 95% CI, 157.59, 7888.28; *p* < 0.00001). Of all the comorbidities, the top comorbidities were: cardiovascular disease; chronic kidney disease; diabetes; and hypertension. Nine comorbidities, except chronic kidney disease, showed a significant amount of heterogeneity (I^2^ value ranging from 77% to 100%) present, which was supported by a strong heterogeneity *p*-value (ranging from 0.00001 to 0.01) and high variability in the confidence interval (Table 1). The risk of bias across studies was low to modest (Figure 4).

**Figure 5:**
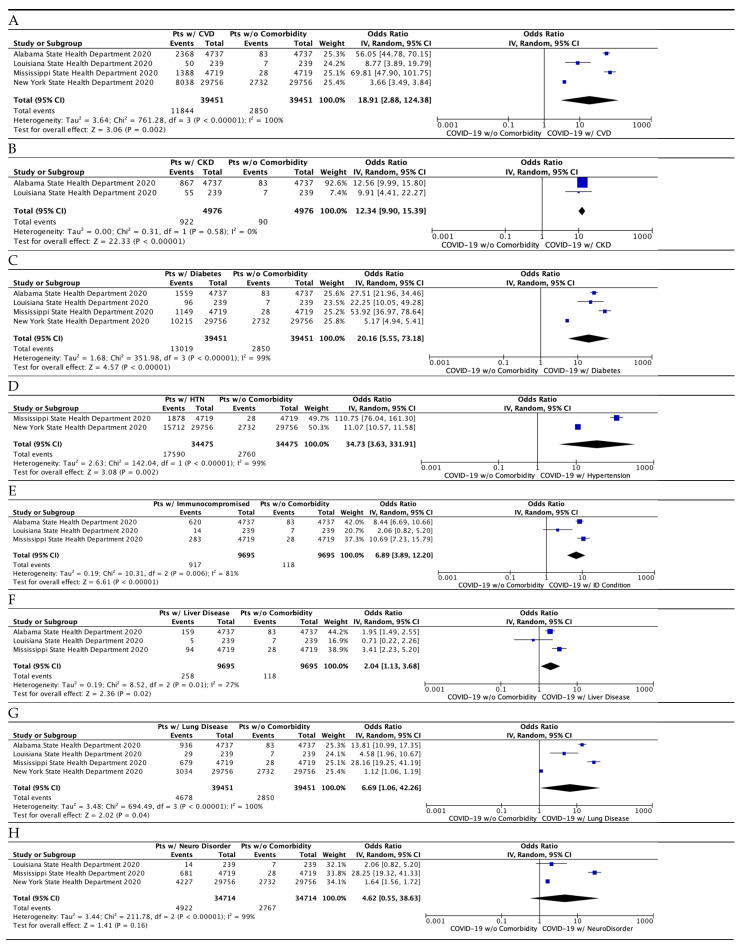

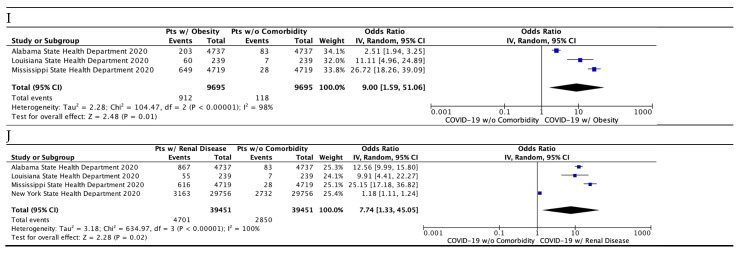
Meta-analysis of the comorbidities with COVID-19. (**A**) Cardiovascular disease (CVD). This odds ratio includes atrial fibrillation, coronary artery disease, hypertension, and stroke from New York’s comorbidity data. (**B**) Chronic kidney disease (CKD). (**C**) Diabetes. (**D**) Hypertension. (**E**) Immunocompromised condition. (**F**) Liver disease. (**G**) Lung disease. This odds ratio includes chronic lung disease from Alabama’s data and COPD from New York’s data. (**H**) Neurological Disease. This odds ratio includes Alzheimer’s disease and stroke data from New York. (**I**) Obesity. (**J**) Renal disease. This odds ratio includes CKD data from Alabama and Louisiana. “Total” columns in the model represent the numerical amount of the total deaths from COVID-19. The “Events” column on the left represents the number of deaths that included the cardiovascular disease comorbidity in that respective state. The “Events” column on the right represents the number of deaths without a comorbidity in that respective state.

As shown in Figure 5A, cardiovascular disease has a high risk of death due to covid-19 (odds ratio 18.91; 95% CI 2.88–124.38; *p* = 0.002). Hypertension is often coupled with cardiovascular disease, and when we combined both hypertension and cardiovascular disease, the risk was doubled (odds ratio, 40.70; 95% CI 27.06, 61.21; *p* < 0.00001). Figure 5B shows the chronic kidney disease (CKD) comorbidity data (odds ratio 12.34; 95% CI 9.90–15.39; *p* < 0.00001; Figure 5B). Importantly, no heterogeneity (I^2^ = 0%) was present in CKD, which is supported by the weak *p*-value (*p* = 0.58) and significant overlap of confidence intervals between the two studies. The lack of heterogeneity in CKD may warrant the use of the fixed-effects method; however, the fixed-effects method provided the result (odds ratio 12.34; 95% CI 9.90–15.39; *p* < 0.00001), which was identical to that obtained by the random-effects model. 

Figure 5C shows the diabetes comorbidity data (odds ratio 20.16; 95% CI 5.55–73.18; *p* < 0.00001). Figure 5D shows the hypertension analysis containing comorbidity data (odds ratio 34.73; 95% CI 3.63–331.91; *p* = 0.002). 

Figure 5E shows the immunocompromised condition comorbidity data (odds ratio, 6.89; 95% CI 3.89, 12.20; *p* < 0.00001); Figure 5F shows the liver disease comorbidity data (odds ratio, 2.04; 95% CI 3.89, 12.20; *p* = 0.02); and Figure 5G shows the lung disease comorbidity data (odds ratio, 6.69; 95% CI 1.06, 42.26; *p* = 0.04). The effect of lung disease as a type of comorbidity may have an overlapping, modest effect on death. Neurological condition (Figure 5H) was not significantly different from no comorbidity (odds ratio, 5.41; 95% CI 0.55, 38.63; *p* = 0.07). It is worth noting that, in New York, dementia is one of the leading comorbidities of COVID-19 deaths, which will require a more specific detailed analysis. Obesity (Figure 5I) had an estimate of 9 (95% CI 1.59, 51.06; *p* = 0.01). The renal disease (Figure 5J) had an estimate of 7.74 (*p* = 0.02). A significant amount of heterogeneity (I^2^ > 77%; *p* < 0.01) was present in this analysis, except for chronic kidney disease (I^2^ = 0%, heterogeneity *p*-value = 0.58).

## 4. Discussion

Age-related health conditions including cardiovascular disease, hypertension, and type 2 diabetes are common in the aging population [4,18,19,20,21,22,23] and have been implicated in late-life diseases such as Alzheimer’s disease [24,25]. The centers for disease control and prevention (CDC) have listed comorbidities with the risk of severe illness from COVID-19, including the comorbidities that are common with increasing age (age-related comorbidities) (e.g., heart conditions, hypertension, and type 2 diabetes) and other comorbidities (e.g., smoking, lung disease, and genetic conditions such as down syndrome and sickle cell disease). Most of the previous studies on comorbidities were based on data from other countries. Using the data available from the US, we estimated the odds ratios of comorbidities within the US population to assess their impact on mortality from COVID-19. We found age-related comorbidities to be the major conditions: cardiovascular disease, chronic kidney disease, diabetes, and hypertension. When cardiovascular disease and hypertension were combined, the odds ratio of mortality became 40 times higher than without a comorbidity. The risks of mortality from COVID-19 in patients with medical comorbidities appear to be much higher than previous studies that used non-US data. For example, the studies about hypertension to which the CDC refers consist most, if not all, of the non-US data (cdc.gov accessed on March 2021). The odds ratios were much higher in the US (odds ratio 34.7; this study) compared to those in studies conducted in non-US countries (odds ratios 2.3–6.5 in non-US countries), e.g., [18,19,20,21], which may imply a variation among countries. In addition to the influence by the control population discussed below (also see the Section of Introduction), the variation in the odds ratios may also be contributed by multiple factors, including demography, methodology, testing procedures, and reporting standards, among others. Thus, a more comprehensive study remains to be done with data from more US states once they become available.

We organized the comorbidity categories with consideration given to the varied definitions of categories between organizations and state reports. In the study, we followed the CDC categories whenever possible. Adjustments of the categories for the purposes of analysis in this study are described in the Results and Methods sections. We also noted that the CDC has a detailed list of diseases contributing to cause of death, which is dominated by “pneumonia and influenza” and “respiratory failure” in the category [15]. In our analysis, we used pre-existing comorbidities present in the population of the study. We tried to avoid skewed results by using the inverse variance method of data analysis to ensure that the data from New York did not overpower data from the other states. Despite the adjustments, the odds ratios were surprisingly higher than those estimated based on previous research. Thus, it is unlikely that the higher odds ratios are influenced by the data from New York or another US state. This study showed that most (92.8%) of the COVID-19 deaths were expected to have a pre-existing comorbidity. Under the condition, comparing with and without a particular comorbid disease would result in comparing the disease and the control population, which likely includes other diseases with high mortality risks, resulting in the underestimation of the mortality odds ratios. For example, comparing with and without diabetes, the latter of which would include hypertension and other comorbidities with high mortality risks, would result in an underestimation of the mortality risk of diabetes. Thus, we used the control population with no comorbidity that avoids the underestimation of the mortality rate in this COVID-19 analysis. 

Most of the comorbidities showed significantly higher mortality as measured by the odds ratio. Comorbidities that significantly increase the risk of a severe COVID-19 include cardiovascular disease, chronic kidney disease, diabetes, hypertension, immunocompromised condition, liver disease, lung disease, obesity, and renal disease. We could not find a significant increase in risk due to neurological conditions. Heterogeneity was significantly high for nine comorbidities, except for chronic kidney disease. We could not assess the mortality of dementia in this study, since the other US states did not report the conditions. Similarly, a recent retrospective cohort study suggests that dementia and neoplastic conditions, including any malignancy and metastatic solid tumor, are significant risks of COVID-19 mortality in the US [26]. Each US state reports dementia and neoplastic conditions differently, which made it difficult to include the conditions in this study. Thus, this study is limited to the resources made available (discussed below). 

We used an adjusted method that has strengths as follows. Firstly, it uses the control population with no comorbidities which avoid bias from the population. The control is particularly important when most of the COVID-19 deaths are associated with one or more comorbidities. Secondly, the mortality data with comorbidities are readily available to the public, which are expected to be less biased. The sample sizes are similar to the previous systematic review and meta-analysis studies [4,18,19,20,21]. Thirdly, it is relatively simple to perform the assessment independent of other factors that are expected to have high variability. Finally, we designed a method that avoids the sources of data ambiguity, such as COVID-19 detection systems and variation in the severity of COVID-19. In contrast, the method has limitations stemming primarily from the availability of the data. It is limited to the resources that have comorbidity data made available to the public, though the limitation is similar to that of the meta-analysis. We were also not able to identify the data about more than one comorbidity, which cannot be assessed in the method. Other limitations have been described above (Methods; Results). Since the method focuses on the mortality data, the outcomes of the results give an overview of the COVID-19 deaths with and without comorbidity. We also note that the comorbidity criteria are inconsistently presented across the states. We propose to have a more unified process of presenting the comorbidities in public health data for collaboration purposes.

## 5. Conclusions

This study is among one of the first US-based studies to show the risk of mortality from COVID-19 in patients who suffer from common comorbidities. We successfully compiled the US COVID-19 mortality data and estimated the risk of death from COVID-19 with and without a comorbidity. This type of study is needed to approximate mortality risk in the context of COVID-19 so that we can better stratify how to address and manage patients with these comorbidities from a public health perspective. Nine out of ten comorbidities analyzed were shown to increase the likelihood of death from COVID-19. We found that, in the US population, the odds ratio for each of these comorbid conditions was higher than that previously seen in the studies predominantly from outside the US. We call for a standardized format and distribution of data to be provided by each state regarding COVID-19 morbidity and mortality, so that a thorough and cohesive analysis of data can occur. We also propose to include a control population with no comorbidity, when possible, to minimize a bias from other health conditions. Altogether, this study gives an awareness about the comorbidities that are more deleterious than previously recognized and provides a call to action for the public health community.

## Figures and Tables

**Table 1:** Summary of the odds ratio outcomes.

Comorbidity Type	OR (95% CI)	*p*	I^2^	Heterogeneity *p*
Cardiovascular Disease	18.91 (2.88, 124.38)	0.002	100%	<0.00001
Chronic Kidney Disease	12.34 (9.90, 15.39)	<0.00001	0%	0.58
Diabetes	20.16 (5.55, 73.18)	<0.00001	99%	<0.00001
Hypertension	34.73 (3.63, 331.91)	0.002	99%	<0.00001
Immunocompromised	6.89 (3.89, 12.20)	<0.00001	81%	0.006
Liver Disease	2.04 (3.89, 12.20)	0.02	77%	0.01
Lung Disease	6.69 (1.06, 42.26)	0.04	100%	<0.00001
Neurological Disease	4.62 (0.55, 38.63)	0.16	99%	<0.00001
Obesity	9.00 (1.59, 51.06)	0.01	98%	<0.00001
Renal Disease	7.74 (1.33, 45.05)	0.02	100%	<0.00001
All Comorbidities Combined	1113.59 (157.59, 7888.28)	<0.00001	100%	<0.00001

Abbreviation: OR (Odds Ratio)**.**

## Data Availability

The data supporting reported results have been made available withing the manuscript.

## Supplementary Materials

The following are available online at https://www.mdpi.com/article/10.3390/idr13030065/s1. Supplementary Material 1: The list of the Public Health weblinks of fifty State and the Washington, D.C. websites searched in this study (as of December 2020), Supplementary Material 2: Demography of the population used in this study, Supplementary Material 3: COVID-19 Hospitalization Data and Rate.
