# Supplementary material for: Unusually High Risks of COVID-19 Mortality with Age-Related Comorbidities: An Adjusted Meta-Analysis Method to Improve the Risk Assessment of Mortality Using the Comorbid Mortality Data
Source: Infect Dis Rep. 2021 Aug 8;13(3):700–11. doi: 10.3390/idr13030065 (PMC8395741; doi:10.3390/idr13030065)
Supplement: Supplementary file 1 [file idr-13-00065-s001.zip › idr-1272219-supplementary.pdf]

Supplementary Material 1

The list of the Public Health weblinks of fifty State and the Washington, D.C. websites searched in this study (as of Dec 2020)

1. Alabama: https://www.alabamapublichealth.gov/index.html
2. Alaska: http://hss.state.ak.us/outage.htm
3. Arizona: https://www.azdhs.gov/
4. Arkansas: https://healthy.arkansas.gov/
5. California: https://www.cdph.ca.gov/
6. Colorado: https://cdphe.colorado.gov/
7. Connecticut: https://portal.ct.gov/dph
8. Delaware: https://www.dhss.delaware.gov/dhss/dph/index.html
9. Washington DC: https://dchealth.dc.gov/
10. Florida; http://www.floridahealth.gov/
11. Georgia: https://dph.georgia.gov/
12. Hawaii: https://health.hawaii.gov/
13. Idaho: https://healthandwelfare.idaho.gov/
14. Illinois: https://www.dph.illinois.gov/
15. Indiana: https://www.in.gov/isdh/
16. Iowa: https://idph.iowa.gov/
17. Kansas: https://www.kdheks.gov/
18. Kentucky: https://chfs.ky.gov/agencies/dph/Pages/default.aspx
19. Louisiana: https://ldh.la.gov/
20. Maine: https://www.maine.gov/dhhs/
21. Maryland: https://health.maryland.gov/pages/home.aspx
22. Massachusetts: https://www.mass.gov/orgs/department-of-public-health
23. Michigan: https://www.michigan.gov/mdhhs
24. Minnesota: https://www.health.state.mn.us/
25. Mississippi: https://msdh.ms.gov/
26. Missouri: https://health.mo.gov/index.php
27. Montana: https://dphhs.mt.gov/
28. Nebraska: dhhs.ne.gov/Pages/default.aspx

29. Nevada: http://dpbh.nv.gov/
30. New Hampshire: https://www.dhhs.nh.gov/
31. New Jersey: https://www.nj.gov/health/
32. New Mexico: https://www.nmhealth.org/
33. New York: https://health.ny.gov/
34. North Carolina: https://www.ncdhhs.gov/
35. North Dakota: https://www.health.nd.gov/
36. Ohio: https://odh.ohio.gov/wps/portal/gov/odh/home
37. Oklahoma: https://oklahoma.gov/health.html
38. Oregon: https://www.oregon.gov/oha/ph/pages/index.aspx
39. Pennsylvania: https://www.health.pa.gov/Pages/default.aspx
40. Rhode Island: https://health.ri.gov/
41. South Carolina: https://scdhec.gov/
42. South Dakota: https://doh.sd.gov/
43. Tennessee: https://www.tn.gov/health.html
44. Texas: https://dshs.state.tx.us/
45. Utah: https://health.utah.gov/
46. Vermont: https://www.healthvermont.gov/
47. Virginia: https://www.vdh.virginia.gov/
48. Washington: https://www.doh.wa.gov/
49. West Virginia: https://dhhr.wv.gov/bph/Pages/default.aspx
50. Wisconsin: https://www.dhs.wisconsin.gov/
51. Wyoming: https://health.wyo.gov/

Supplementary Material 2

Demography of the population used in this study.

State Health Department	# of Deaths	Gender			Race/Ethnics							Access Date
		Male	Female	Unknown	White	Black	Asian	American Indian or Alaska Native	Unknown	Other	Hispanic	
Alabama	4,737*	2,487 (53%)	2,240 (47%)	10 (0.2%)	2,723 (58%)	1,544 (33%)	18 (0.4%)	NA	307 (7%)	146 (3%)	**	12/29/20
Louisiana	239	NA	NA	NA	NA	NA	NA	NA	NA	NA	NA	12/17/20
Mississippi	4,719	NA	NA	NA	2,441	1,964	15	94	125	80	**	12/29/20
New York	29,756	16,849 (57%)	12,898 (43%)	9 (0.0%)	NYC 27% NYS Excluding NYC 66% ***	NYC 28% NYS Excluding NYC 15% ***	NYC 7% NYS Excluding NYC 4% ***	NA	NA	NYC 4% NYS Excluding NYC 3% ***	NYC 34% NYS Excluding NYC 12% ***	12/29/20

Abbreviations: NA (Not available), NYC (New York City), NYS (New York State).
* The Alabama percentages by race add up more than 100%. It is a possibility of patients in two or more race.
** Alabama and Mississippi label Hispanic as an ethnicity, but not a race. Alabama reports 109 of the total deaths were of Hispanic ethnicity and Mississippi reports 50 deaths were of Hispanic ethnicity.
*** NYC data and NYS excluding NYC data were available as percentages shown. The numbers were not available.

Supplementary Material 3

COVID-19 Hospitalization Data and Rate.

State	CDC Hospitalization Rate per 100K patients (12/26/20)		COVIDtracking.com # of Hospitalized Patients		University of Minnesota COVID-19 Hospitalization Tracking Project				Total Deaths
	Cumulative	Weekly	Cumulative Hospitalizations	Actual # of Hospitalizations	Cumulative Hospitalizations	Cumulative Hospitalizations per 100k adults (20+ y.o.)	Current # of Hospitalizations	Current Hospitalizations per 100k adults (20+ y.o)	
Alabama	NA	NA	33,452 (12/29/20)	2,804 (12/29/20)	33,452 (12/29/20)	911 (12/29/20)	2,804 (12/29/20)	76.39 (12/29/20)	4,737
Louisiana	NA	NA	NA	1,355 (3/31/20)	NA	NA	1,135 (3/31/20)	32.92 (3/31/20)	239
Mississippi	NA	NA	8,145 (12/28/20)	1,325 (12/28/20)	8,145 (12/28/20)	371 (12/28/20)	1,340 (12/28/20)	61.04 (12/28/20)	4,719
New York	290*	38.7*	NA	7,814 (12/29/20)	136,825 (12/29/20)	912* (12/29/20)	7,892* (12/29/20)	52.6 (12/29/20)	29,756

*, Discrepancies found in the New York data possibly due to the different data administration, recording and reporting.

The data from the three resources are listed, including CDC [1], COVIDtracking.com [2] and University of Minnesota COVID-19 Tracking Project [3]. The dates of the data update are shown in the Table. The data were last updated on the dates shown in the table and accessed on 7/12/2021. Abbreviation, NA (data not available); y.o. (years old). No comorbidity data were associated with the numbers indicated.

References:

1. The Centers for Disease Control and Prevention (CDC). COVID-19 Hospitalizations. Available online: https://gis.cdc.gov/grasp/covidnet/covid19_3.html (accessed on 11 July 2021).
2. The COVID Tracking Project. The Data. Available online: https://covidtracking.com/data (accessed on 11 July 2021).
3. Carlson School of Management. COVID-19 Hospitalization Tracking Project. Available online: https://carlsonschool.umn.edu/mili-misrc-covid19-tracking-project (accessed on 11 July 2021).
